# Image denoising with morphology- and size-adaptive block-matching transform domain filtering

**DOI:** 10.1186/s13640-018-0301-y

**Published:** 2018-07-20

**Authors:** Yingkun Hou, Dinggang Shen

**Affiliations:** 1 School of Information Science and Technology, Taishan University, Taian 271000, China; 2 Department of Radiology and Biomedical Research Imaging Center, University of North Carolina at Chapel Hill, Chapel Hill, NC 27599, USA; 3 Department of Brain and Cognitive Engineering, Korea University, Seoul 02841, South Korea

**Keywords:** Block-matching, Size-adaptive filtering, Morphological component, Image denoising

## Abstract

BM3D is a state-of-the-art image denoising method. Its denoised results in the regions with strong edges can often be better than in the regions with smooth or weak edges, due to more accurate block-matching for the strong-edge regions. So using adaptive block sizes on different image regions may result in better image denoising. Based on these observations, in this paper, we first partition each image into regions belonging to one of the three morphological components, i.e., contour, texture, and smooth components, according to the regional energy of alternating current (AC) coefficients of discrete cosine transform (DCT). Then, we can adaptively determine the block size for each morphological component. Specifically, we use the smallest block size for the contour components, the medium block size for the texture components, and the largest block size for the smooth components. To better preserve image details, we also use a multi-stage strategy to implement image denoising, where every stage is similar to the BM3D method, except using adaptive sizes and different transform dimensions. Experimental results show that our proposed algorithm can achieve higher PSNR and MSSIM values than the BM3D method, and also better visual quality of denoised images than by the BM3D method and some other existing state-of-the-art methods.

## 1 Introduction

Image denoising, as a basic topic of pattern recognition and computer vision, has been studied for many years. However, there are still many new methods and algorithms that have been proposed in recent years. Particularly, the non-local method has become a mainstream method. For example, Buades et al. [1] proposed a novel method for image denoising, which was named as non-local means (NL_means). Afterwards, many other non-local methods have been proposed, in which the block-matching 3D (BM3D) [2] transform domain filtering method is the most successful. BM3D is the current state-of-the-art image denoising method [3, 4], and, as a special non-local image denoising model, can achieve very precise image denoising results.

Recently, a variety of new image denoising methods have also been proposed, but very few approaches can perform better than BM3D. Many of these methods are based on the non-local idea, i.e., using similar image blocks (or patches) to explore new image denoising methods. For example, Zhang et al. [5] proposed a two-stage principal component analysis (PCA) on local pixel grouping, with its local pixel grouping achieved by block matching. Although this method successfully combined the classical PCA with nonlocal idea and achieved better results than those traditional local methods, the denoising performance of this method is still lower than the BM3D method. Rajwade et al. [6] used the higher order singular value decomposition (HOSVD) for image denoising, by modifying the 3D transform in BM3D to HOSVD. For the color images with strong noise, HOSVD method can slightly perform better than BM3D, however, this method is not better than BM3D for the gray images or lower noise level situations. Papyan et al. [7] proposed a multi-scale patch-based method by improving the expected patch log likelihood (EPLL) method [8], this method can achieve better subjective visual quality of the denoised images with less artifacts, it can also be applied to other image processing problems, such as deblurring and super-resolution. There are also many other patch-based image denoising methods [9–18] in the literature. Some of them are improved non-local methods, while others are improved BM3D methods. Except patch-based methods, dictionary-learning based methods [19–28] can also achieve good image denoising results. For example, Dong et al. [29] proposed a combined *non-local* and *bilateral variance estimation* method, it provided a conceptually simple interpretation from a bilateral variance estimation perspective to further proposed a spatially adaptive iterative singular-value thresholding (SAIST), this method can achieve better denoised results in higher noise level situations, but it is only partially superior to BM3D. Most recently, there have been some new denoising methods developed. For example, Lebrun et al. [30] proposed a blind denoising algorithm, which can automatically estimate image noise. Ghimpeteanu et al. [31] proposed a decomposition framework for image denoising, and can better preserve image geometry (i.e., directions of gradients and level lines), when used with some existing denoising methods, such as NL_means and BM3D. Romano et al. [32] proposed a boosting strategy, i.e., employing a “SOS” procedure, to improve image denoising performance of some existing image denoising methods. But this method can only improve very little on the BM3D method, Romano et al. [33] also proposed a patch similarity measurement problem and used it to three kinds of image processing application. J. Mairal et al. [34] proposed non-local sparse models (LSSC, for learned simultaneous sparse coding) for image restoration which can better preserve image details than BM3D method on image denoising. S. Gu et al. [35] proposed a weighted nuclear norm minimization (WNNM)-based image denoising method, which achieved nice results especially on some texture-rich images, for example, House, and Barbara, however, this method usually produce some artifacts in some parts of the denoised images, especially in higher level noise situations.

BM3D method’s success in image denoising mainly comes from the two main characteristics: (1) natural images usually hold a large number of similar image blocks, and (2) contents in small image block is often locally highly correlated. Based on these two characteristics, grouping operation assembles the highly correlated blocks into each slide of the 3D matrix, and then sparse representation of real signal can be achieved by the de-correlation 3D transform. Due to the sparsity, effective image denoising can be realized just by the coefficients shrinkage with hard threshold. BM3D filtering is a powerful denoising method, and its denoising results are often much better than most existing denoising algorithms. Moreover, the BM3D-based denoising effects will be especially prominent when many easy matching blocks (such as with textures and/or contours) can be found. However, the basic assumption of high correlation of local image contents in the fixed-size square image block is not always holding. For example, if some image blocks contain weak image details, singularity, and sharp edges, the *non-adaptive* transform usually cannot obtain effective sparse representation. Therefore, in this case, the BM3D filtering may introduce some artifacts, and denoising is often not very effective. But this kind of image contents is often the most important part of the human visual attention.

On the other hand, shape-adaptive discrete cosine transform (SA-DCT) [36] is another type of image denoising methods, which uses neighborhoods with adaptive shapes to local image contents. Thus, in each shape-adaptive neighborhood, the discrete cosine transform can achieve sparse representation of real signal, effectively shrinking the transform domain coefficients and achieving image denoising. Due to the adaptability of neighborhood to local image details, SA-DCT can better preserve image details after denoising. However, SA-DCT has a disadvantage, e.g., it can fail in the texture-rich regions since local homogeneity in these regions is very limited. In addition, SA-DCT is a kind of local filter, and thus cannot make full use of repetitive structures or patterns in the natural images.

In order to achieve better results for image denoising, a shape-adaptive BM3D (SA-BM3D) [37] has been proposed by combining the advantages of both BM3D and SA-DCT. Specifically, SA-BM3D groups similar shape- adaptive neighborhoods (such as image patches), instead of shape-fixed image blocks in the BM3D method. In this way, the adaptation of non-local model, and also the local image characteristics, can be simultaneously used by particularly improving the spatial correlation within each image patch. As each image patch is not necessarily square, the orthogonal wavelet transform cannot be directly applied to the shape-adaptive image patch. Therefore, SA-DCT is first used on each shape- adaptive image patch, and then one-dimensional orthogonal wavelet transform is performed in the third dimension, followed by shrinking the transform coefficients with hard thresholding or Wiener filtering to finally achieve image denoising.

Another improvement to BM3D is, namely, *BM3D shape adaptive principal component analysis* (BM3D-SAPCA) [38], by combining the advantages of the SA-BM3D and the principal component analysis (PCA) methods. Note that this improved method changed DCT in SA-BM3D to PCA, by using eigenvalue decomposition of each image patch to get a PCA basis for selecting some eigenvectors with eigenvalues greater than a certain threshold (determined by noise level) as principal components. As a result, the whole 3D transform is changed to a new kind of separable transform combinations, i.e., performing PCA on each image patch, and then performing one-dimensional orthogonal transform on the third dimension. BM3D-SAPCA achieved better denoising results than the classical BM3D and SA-BM3D, by preserving better image details and introducing less image artifacts. Besides, Chen et al. [39] proposed a bounded BM3D, which has a little bit improvement on BM3D only for relatively higher noise level. Zhong et al. [40] proposed modified BM3D algorithm for image denoising using non-local centralization prior; this method removed the 1D transform inter-blocks and utilized a prior to improve the BM3D method; however, the denoised results are only partially better than BM3D method.

The successes of both BM3D-SAPCA and SA-BM3D are primarily by the use of shape-adaptive image patches/ neighborhoods. But, the procedure for computing local adaptive shapes is relatively complex. For example, PCA often needs greater calculation time than the twodimensional orthogonal transformation, and thus the whole operation of BM3D-SAPCA is time-consuming. Most importantly, it is difficult to make shapes adaptive, when the noise level is relatively high. To address these issues, in this paper, we propose an improved block-size-adaptive BM3D method for image denois- ing. First, DCT is performed on the reference image block before conducting the block matching. Then, all image blocks can be divided into three morphological components (namely, smooth, texture, and contour regions) based on the regional energy of alternating current (AC) component in the DCT coefficients. For different morphological component, the size of the reference image block will be enlarged or reduced. For example, the size for smooth-component image block will be enlarged appropriately, and the size for the contour-component image block will be reduced, while the size for the texture-component image block will be kept as the original size. Experimental results show that our proposed method can achieve better image denoising results than both BM3D and BM3D-SAPCA, in terms of PSNR and MSSIM values, and can also preserve better image details and introduce less image artifacts.

## 2 Morphological component representation in image

In recent years, a morphological component analysis method [41] has been proposed and widely used in image processing. The main idea of morphological component analysis is to divide the image contents into different components, such as smooth, texture, and contour. As DCT is a tool that can effectively depict periodic signals, we perform DCT on image blocks and then classify the image blocks into different morphological components according to their respective energies of the alternating current (AC) coefficients.

### 2.1 2D discrete cosine transform (DCT)

DCT, normally used in signal processing and image processing, is often used for compression of signal and image data (including *still images* and *motion images*). This is because DCT has relatively strong “energy concentration” features, i.e., the energy of most natural images is concentrated in the low frequency part after DCT. A 2D DCT is defined as

(1) $$F(u,v)=c(u)c(v)\sum_{i=0}^{N-1}\sum_{j=0}^{N-1}f(i,j)\cos\left[\frac{(i+0.5)\pi }{N}u\right]\;\;cos\left[\frac{(j+0.5)\pi }{N}v\right]$$

where $c(u)=\{\begin{array}{l} \sqrt{\frac{1}{N}},u=0\\ \sqrt{\frac{2}{N}},u\neq 0 \end{array},\;\text{and}\;c(v)=\{\begin{array}{ll} \sqrt{\frac{1}{N}}, & v=0\\ \sqrt{\frac{2}{N}}, & v\neq 0 \end{array}$.

For faster and more convenient implementation of DCT on each image block, the BM3D method first generates a DCT forward transform matrix *T*_*for*_ and also an inverse transform matrix *T*_inv_, where $T_{\mathrm{for}}(i,j)=c(i)\cos\left[\frac{(j+0.5)\pi }{N}i\right]$ and $T_{\text{inv}}=T_{\text{for}}^{T}$.

The forward transform on each image block *B* is defined as the following:

(2) $$\hat{B}=T_{\text{for}}\cdot B\cdot T_{\text{for}}^{T}$$

The inverse transform is similar to the forward one, i.e.,

(3) $$\hat{\hat{B}}=T_{\text{inv}}\cdot \hat{B}\cdot T_{\text{inv}}^{T}$$

A 8 × 8 DCT forward transform matrix is given as the following:

(4) $$T_{\text{for}}=\left(\begin{array}{cccccccc} 0.3536 & 0.3536 & 0.3536 & 0.3536 & 0.3536 & 0.3536 & 0.3536 & 0.3536\\ 0.4904 & 0.4157 & 0.2778 & 0.0975 & -0.0975 & -0.2778 & -0.4157 & -0.4904\\ 0.4619 & 0.1913 & -0.1913 & -0.4619 & -0.4619 & -0.1913 & 0.1913 & 0.4619\\ 0.4157 & -0.0975 & -0.4904 & -0.2778 & 0.2778 & 0.4904 & 0.0975 & -0.4157\\ 0.3536 & -0.3536 & -0.3536 & 0.3536 & 0.3536 & -0.3536 & -0.3536 & 0.3536\\ 0.2778 & -0.4904 & 0.0975 & 0.4157 & -0.4157 & -0.0975 & 0.4904 & -0.2778\\ 0.1913 & -0.4619 & 0.4619 & -0.1913 & -0.1913 & 0.4619 & -0.4619 & 0.1913\\ 0.0975 & -0.2778 & 0.4157 & -0.4904 & 0.4904 & -0.4157 & 0.2778 & -0.0975 \end{array}\right)$$

**Fig. 1:**
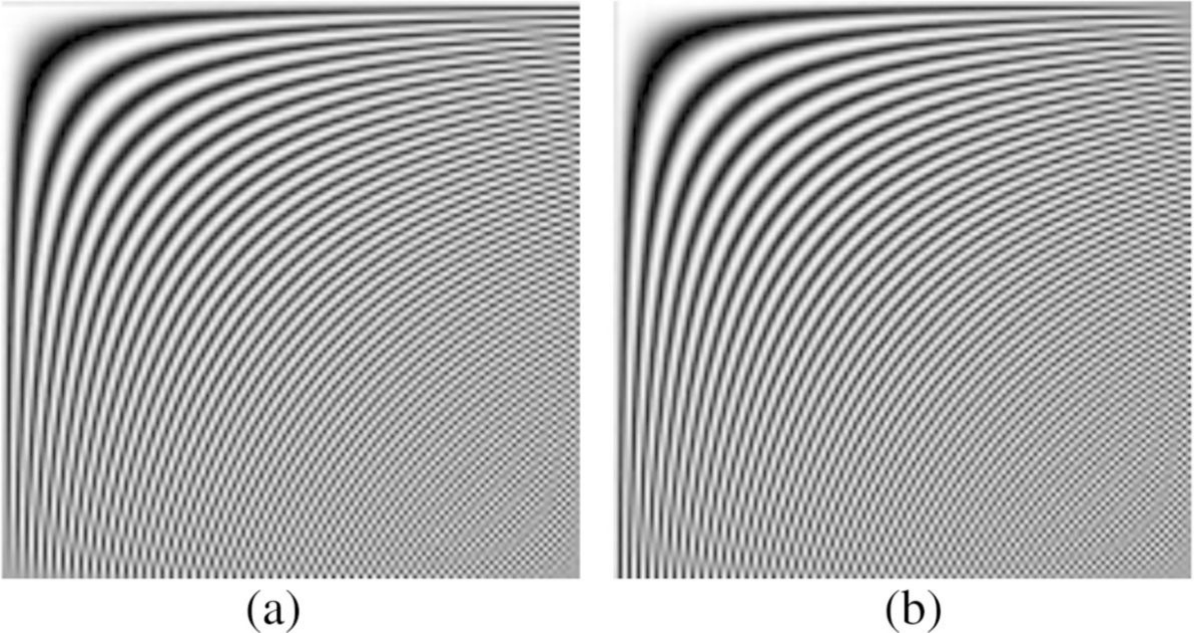
Ilustration of DCT transform matrices. **a** Forward transform matrix, and **b** inverse transform matrix

For visual inspection, the 128 × 128 *T*_for_ and *T*_inv_ matrices can also be displayed as images in Fig. 1, respectively.

### 2.2 Classification of morphological components in 2D image

In this section, we give a classification method for determining the morphological component of each image block. Specifically, we first implement the DCT forward transform on a given image block *B*_*R*_,

(5) $${\hat{B}}_{R}=T_{for}\cdot B_{R}\cdot T_{\text{for}}^{T}$$

Then, we compute the AC energy of the transform spectrum ${\hat{B}}_{R}$ by the following equation:

(6) $$E_{\text{AC}}=\sum_{i=1}^{N}\sum_{j=1}^{N}\left|{\hat{B}}_{R}(i,j)\right|-\left|{\hat{B}}_{R}(1,1)\right|$$

Finally, we can classify the morphological component of an image block as follows:

(7) $$\{\begin{array}{ll} C_{\text{contour}}, & \;if\;E_{\text{AC}}-c\sigma \geq K_{1}\\ C_{\text{texture}}, & if\;K_{2}\leq E_{\text{AC}}-c\sigma <K_{1}\\ C_{\text{smooth}}, & \;if\;E_{\text{AC}}-c\sigma <K_{2} \end{array}$$

Where K and *K*_*2*_ are the two empirical values used to classify the morphological component. In this paper, we use the following method to define the parameter *c*: (1) add different-level Gaussian white noises to a constant value 8 × 8 image block, (2) perform DCT transform on this noisy image block, and (3) use Eq. (6) to calculate the energy *E*_*AC*_, we found that *E*_*AC*_ has proportional relationship with the standard deviation of noise, σ, by many experiments, the relationship between them are as follows

(8) $$E_{\text{AC}}=c\sigma$$

Thus, we can get value for *c* as *c* = 0.18.

## 3 Influence of morphological components on image denoising with BM3D

The number of similar blocks is an important factor on non-local image denoising problem, the accuracy of block matching in transform domain filtering is also critical in influencing the denoising performance. Romano and Elad [33] and Levin et al. [42] respectively analyzed the influence of block size on image block matching accuracy and denoising performance; they theoretically discussed the effect of block size on the accuracy of image block matching. In the classic BM3D algorithm, the size of the image block is fixed. However, for image blocks with different morphological components, using fixed block size could limit the accuracy of block matching results or the denoising ability. Actually, the bigger block size has the stronger denoising ability in advance of the accurate block matching; however, image blocks with contour component are difficult to get higher accuracy of block matching when bigger image blocks are used than those image blocks in smooth areas. So we had better use bigger blocks in smooth areas but smaller blocks in texture or contour areas. This is consistent with human visual perception, and inspired us to use different block size in block matching, according to different morphological components.

**Fig. 2:**
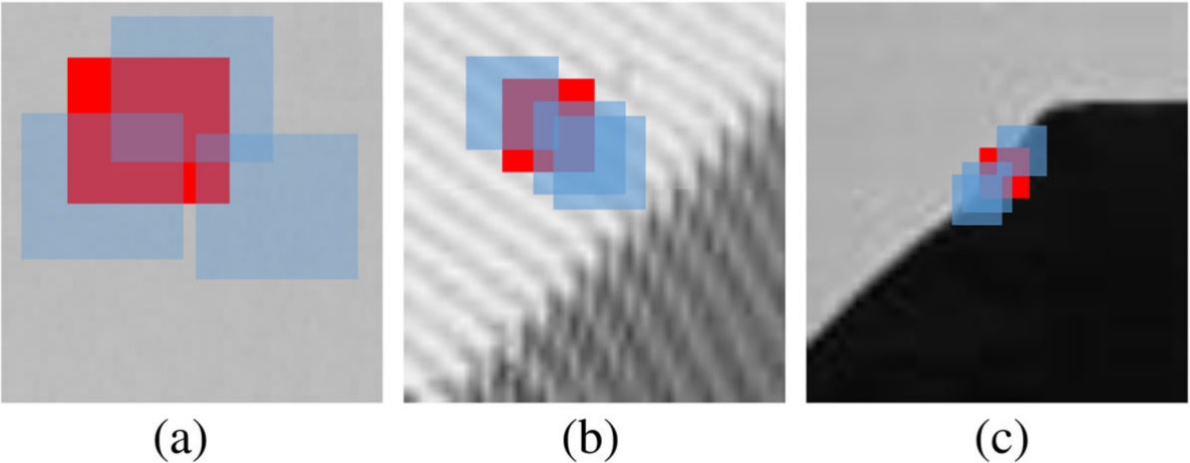
III ustration of image blocks with three morphologlcal components, i.e., with **a** smooth, **b** texture, and **c** contour components, respectively

**Fig. 3:**
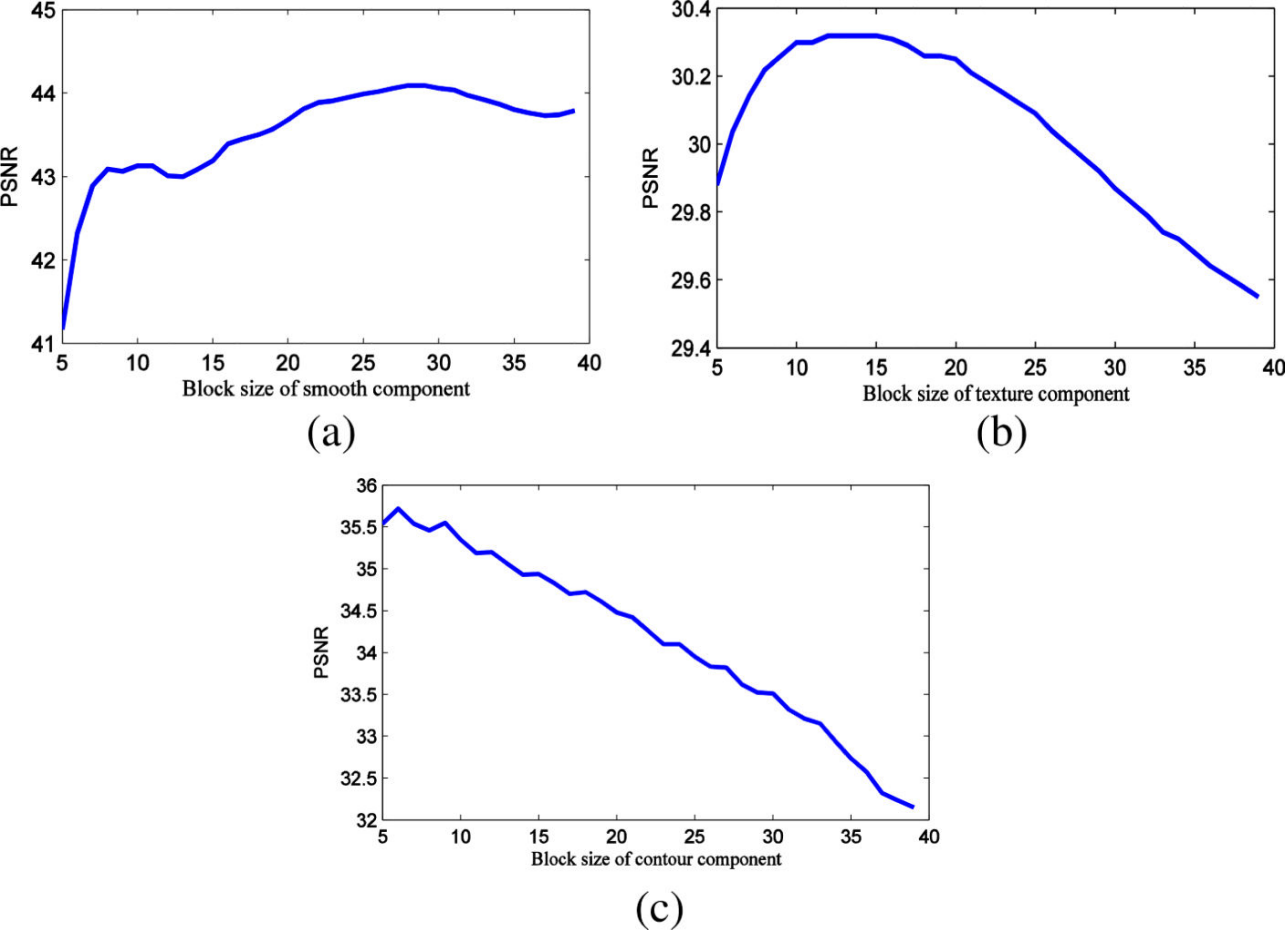
Changes of denoising result *with respect to* the use of different block size, for three image blocks with **a** smooth component, **b** texture component, and **c** contour component, respectively

To verify this idea, we extract three image blocks with different morphological components from House, Barbara, and Cameraman images, and then perform denois- ing with BM3D, as shown in Fig. 2. Later, we first add the Gaussian white noise with the standard deviation 25 to these three image blocks, and then use BM3D to denoise them. In the process of denoising, all other parameters remain the same, by changing only the block size, to fairly compare the denoising results. From the PSNR values of denoised image blocks shown in Fig. 3, we can draw the two following observations: (1) under the same noise level, the denoising result of the image block with smooth component is the best, followed by the image block with contour component and the image block with texture component; and (2) the image block with smooth component can usually achieve better denoised results using large block size, while the image block with texture component needs median block size and the image block with contour component needs small block size. These two observations, especially the second observation, inspire us to adaptively select block size according to the morphological components, when performing block-matching filtering for the image denoising.

## 4 Methods

It is worth noting that the 3D transform in BM3D algorithm is separable. For example, we can first implement 2D transform on each block in every image block group, which is obtained by block matching, and then implement the 1D transform along the third dimension. Since the 2D transform is still local, there exist inevitable disadvantages of local transform, such as introduction of artifacts as well as blurry image edges after denoising. If an enough number of similar noisy image blocks can be obtained, the 1D transform can help denoise the noisy image very well. In particular, no transformation is required, as we can simply average those similar blocks in the case of added white Gaussian noise (AWGN) and achieve the ideal denoised results. Unfortunately, in reality, there are not many similar image blocks in the single image. Therefore, to avoid performing 2D transform on each image block, we can iteratively perform 1D transforms, thus better preserving edges and introducing less artifacts.

**Fig. 4:**
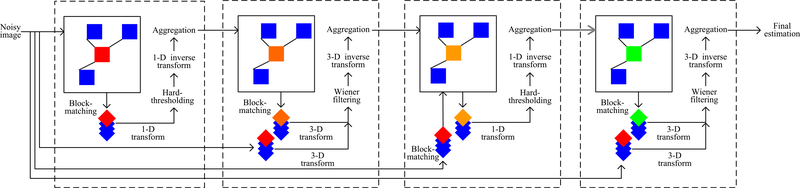
The flowchart of the proposed algorithm

On the other hand, however, there are also two problems of iteratively performing 1D transforms. The first problem is that the image blocks are not completely the same. Even if we just use 1D transform, the image edges are only smoothed to a certain degree. The second problem is that some isolated strong noises will be retained if we just use 1D transform to denoise the image, since the transformed coefficient magnitude of strong noise will be very large, i.e., even larger than the coefficient magnitude of real signal. Thus, when we use a hard threshold to shrink the transformed coefficients, we cannot remove those strong noises. To solve these two problems and also achieve better image denoising results, we propose an improved BM3D algorithm. The respective improvements include (1) the use of adaptive block size, and (2) the use of multi-stage strategy. This improved BM3D algorithm includes four steps, as shown in Fig. 4. As for the multi-stage strategy, we have presented more details in a previous conference paper [43].

### 4.1 Step 1: block matching 1D (Haar) transform domain filtering

In the original BM3D algorithm, the first step is block-matching 3D transform. Since the 2D transform on each block is still the local transform, some artifacts will be introduced after inverse transform. Also, since the transformed coefficient magnitude of strong noise is very large, the hard-thresholding operation can only remove the coefficients of those relatively small noises. To avoid the introduction of artifacts, we can only perform 1D transform along the third dimension, in which this 1D transform is a real non-local transform. So, in this step, we first implement block-matching operation according to the BM3D method, and then perform 1D Haar transform on the third dimension, i.e., the inter-block Haar transform. Note that we will not implement the 2D transform on each block, so we call this step as *block matching 1D transform* (BM1D). Also, since we just implement 1D transform, only a few noises can be separated from the real signal, while many other noises, especially strong noises, are still retained after this step. On the other hand, image edges can be better preserved and few artifacts are introduced.

To further efficiently preserve image edges, we use a perturbation strategy, i.e., we amplify the low frequency coefficients after the 1D transform as follows:

(9) $${\hat{B}}_{G}=T^{-1}\left(\mathrm{shrink}\left(\left[{\left(T\left(B_{G}\right)\right)}_{1}\cdot \gamma ,{\left(T\left(B_{G}\right)\right)}_{2},\cdots ,{\left(T\left(B_{G}\right)\right)}_{K}\right]\right)\right)$$

where (*T(B*_*G*_))_1_ is the low frequency subband of the 1D transform on image block group *B*_*G*_, and γ > 1 is a gain factor, which is used to amplify the low frequency coefficients. *T* and *T*^−1^ are the forward and inverse 1D transforms, respectively. And shrink denotes a hard-thresholding operation. This perturbation strategy has two major functions: (1) it can protect low frequency coefficients without shrinkage, because most coefficients of image edges belong to low frequency subband after the 1D transform, and (2) it can reduce the effect of strong noise in the next step of block-matching, thus improving the block-matching accuracy. On the other hand, however, it will reduce denoising performance. So we next implement BM3D Wiener filtering to further denoise the result of this step.

### 4.2 Step 2: block matching 3D Wiener filtering

We use the resulted image of the first step as reference, and perform empirical Wiener filtering on the original noisy image. The purpose of using BM3D-based Wiener filtering in this step is to enhance weakened image edges after using the first step, as image edges are always weakened to some extent after applying the first step of noise reduction. The following is the empirical Wiener filtering:

(10) $${\text{Shrink}}_{\text{wie}}(\theta )=\frac{\theta \cdot |\hat{\theta }{|}^{2}}{|\hat{\theta }{|}^{2}+\sigma^{2}}$$

where $\hat{\theta }$ is the block matching 3D transformed coefficients of the result image of the first step, θ is the block matching 3D transformed coefficients of the original noisy image, and *σ* is the standard deviation of noise. Actually, we can use a smaller σ in this step to better preserve image edges, the smaller *σ* can obtain the similar effects with the low frequency coefficients perturbation in the first step.

Note that here we use 3D transform, instead of 1D transform in the first step, because using 3D transform and hard-threshold operation can remove strong noises. Of course, 3D transform is not good at preserving image edges than 1D transform. After completing this second step, noises can be further removed partly.

### 4.3 Step 3: size-adaptive block matching 1D transform domain filtering

In this third step, we first perform DCT on each reference block before using the block matching operation, then use Eq. (6) to calculate the AC energy of the transformed coefficients, and finally determine the class of morphological component by Eq. (7), which will be used to adjust the block size according to the descriptions in Section 3. Note that this step is the most critical step in the whole algorithm. This is because, after the second step, there still exist a lot of noises. Moreover, since DCT is operated on each block of BM3D Wiener filtering in the second step, some strong noises become the certain *pseudo textures,* which are no longer subject to Gaussian distribution. Thus, we should not perform block matching on the results produced by the second step. To get rid of these noises with *certain pseudo textures*, we perform block matching on the original noisy image, and then use the block matching results to extract image blocks at the same locations to implement 1D Haar transform among blocks. Finally, we similarly use a hard-threshold operation to remove the noise, which can be effectively done except for few isolated strong noises.

It is worth noting that we use size-adaptive blocks to improve block-matching accuracy. In the smooth region, if we use a small size to perform block matching, the noise, especially isolated strong noises, would influence the block-matching result. In other words, it would result in noise matching, instead of real signal matching. In the contour region, if we use a large size to perform block matching, it would be difficult to obtain ideal contour matching result, as contours are illustrated as strait lines. However, if we use a small size to perform block matching in these contour regions, we can easily obtain accurate contour matching results. In the texture region, the block size is in-between the former two. This is because if the size is too small, the block matching result will be influenced by the noise; while, if the size is too big, it is still difficult to obtain accurate block matching result as the contour region.

### 4.4 Step 4: size-adaptive block matching 3D Wiener filtering

In the fourth step, similar to the third step, we first use Eqs. (5)-(7) to determine the class of morphological component for the reference block, and then adjust the size for the reference block. The rest of the procedure in this fourth step is the same as the classic BM3D Wiener filtering.

**Fig. 5:**
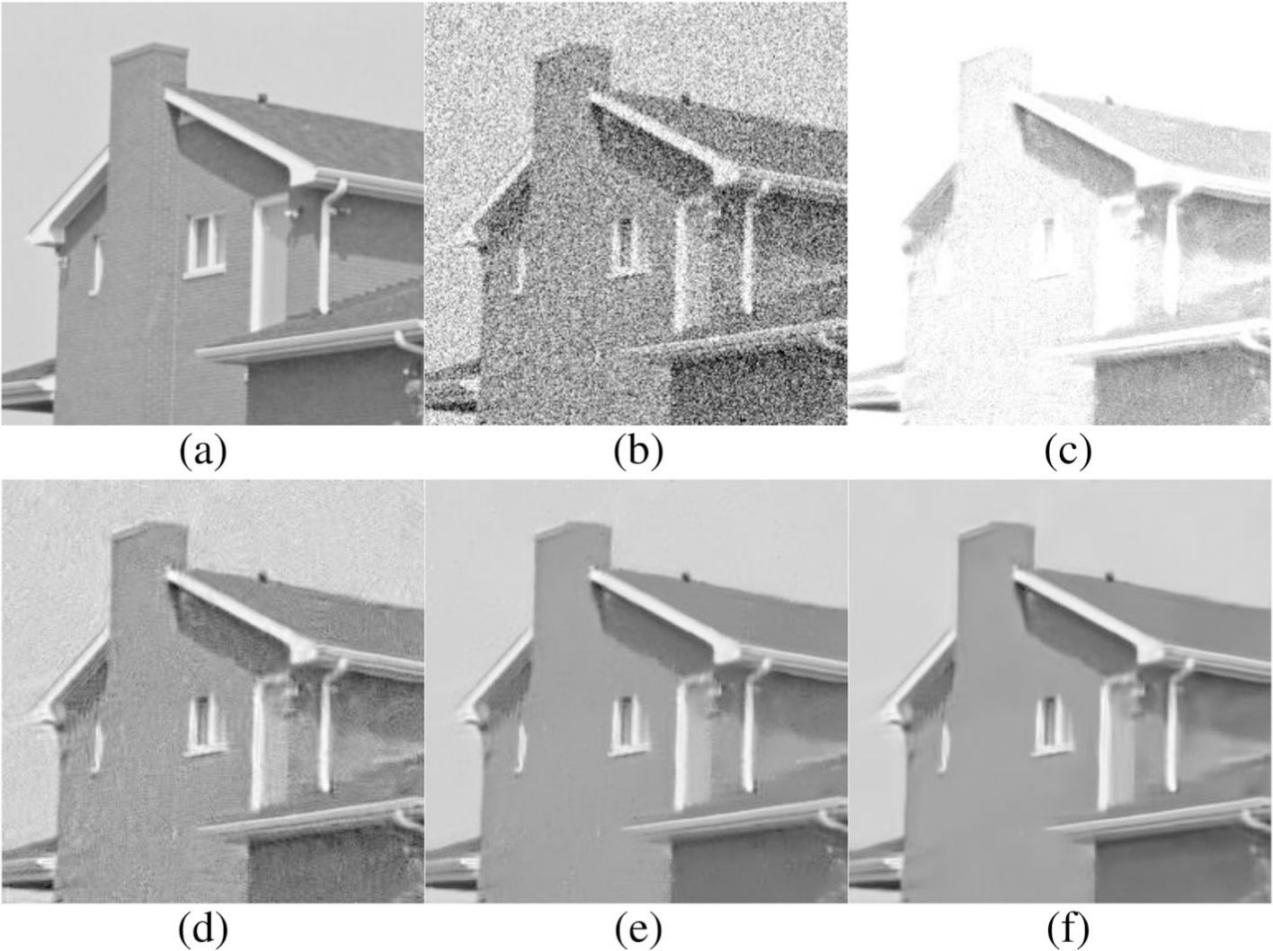
Denoised results of House image at each step of SA-BM1–3D. **a** Original image, **b** noisy image (σ =50), **c** step 1, **d** step 2, **e** step 3, and **f** step 4

After completing the above four steps, we are able to obtain a final denoised image. Notably, all of the first three steps are equivalent to the first step in the original BM3D algorithm, i.e., the basic estimation stage in the original BM3D algorithm. Because we use both 1D and 3D transforms as well as the size adaptive block-matching in the proposed algorithm, we call the proposed algorithm as SA-BM1–3D in the rest of this paper. To better understand the procedure of SA-BM1–3D, we use Fig. 5 to show the results of each step.

To denoise color images, we also transform RGB image to luminance-chrominance image just like BM3D; there are three channels in a luminance-chrominance image, i.e., Y, U, and V, respectively. We also use the opponent color transformation to obtain luminance-chrominance image, its transform matrix refers to [2]. In each step of SA-BM1–3D, we use Y channel to perform block-matching, then apply the block-matching result to other two channels, i.e., U and V. We still use the proposed 4-step algorithm to denoise each channel.

## 5 Experimental results and discussion

### 5.1 Parameter values

In this section, we give all the parameter values for SA-BM1–3D algorithm, determined based on our experiments. The name of each parameter in SA-BM1–3D is summarized below.

#### 5.1.1 Step 1 (ID transform)

*Si* block size, the block size is gradually increased with the noise level becoming higher and higher. The block matching can easily achieve better results as the noise level is low, so we use the smaller size, however, the higher noise level will significantly influence the block-matching accuracy, so we should utilize relatively bigger block size. *N*_1_: the number of blocks in each group, when noise level is low we use small number so as to guarantee the sufficient similarity among the matched blocks to better preserve weak texture and contour after denoising. The relatively bigger number for higher noise level to achieve the better denoising; *T*_1_: hard threshold, we use the same value in this stage, because this stage is just slightly denoising, we need not use different hard threshold whether lower or higher noise level, the common value is enough; γ: perturbation factor, the purpose of this parameter is to reduce the influential of the isolated strong noise, in other words, increase the block matching accuracy in the next step. With the increase of the noise level, the isolated strong noise will also be stronger, so we use the bigger value for higher noise level.

#### 5.1.2 Step 2 (Wiener filtering)

*S*_*2*_*:* block size; *N*_*2*_*:* the number of blocks in each group; *T*_2D_^:^ 2D transform on each block with DCT. All the parameters in this step are the same as BM3D Wiener filtering step, because we implement the same Wiener filtering operation in BM3D method in this step.

#### 5.1.3 Step 3 (size-adaptive ID transform)

S_3_: initial block size, we implement DCT on an initial 8×8 image, then use its AC component to decide the morphological component, then further adjust the block size according different morphological components; *N*_*3*_*:* the number of blocks in each group, the principle is the same as step 1; *T*_2_: hard threshold. Because we utilized bigger block size for higher noise level in step 1, the denoising performance is stronger than the lower noise level ones, so we use the relatively smaller hard threshold for higher noise level situations in this step to better preserve texture or contour details.

#### 5.1.4 Step 4 (size-adaptive Wiener filtering)

*S*_4_: initial block size, the same selection principle as the S_3_; *N*_*4*_*:* the number of blocks in each group, the same principle as the Wiener filtering step in BM3D method; *T*2D^:^ 2D transform on each block with DCT.

Table 1 shows all the parameter values at different noise levels. Three other parameters (not listed in Table 1) are also used in each step, such as (1) *N*_step_=3 for the sliding step size of reference block, (2) *N*_*S*_=39 × 39 for the searching neighborhood size in block matching, and (3) Haar transformation on the third dimension.

Both steps 3 and 4 use adaptive block size, with the initial block size as 8 × 8. Then, we adaptively determine the block size according to the AC energy of DCT coefficients, i.e., using the block size of 17 × 17 for the smooth component, 7×7 for the texture component, and 4×4 for the contour component, respectively.

### 5.2 Experimental results

**Fig. 6:**
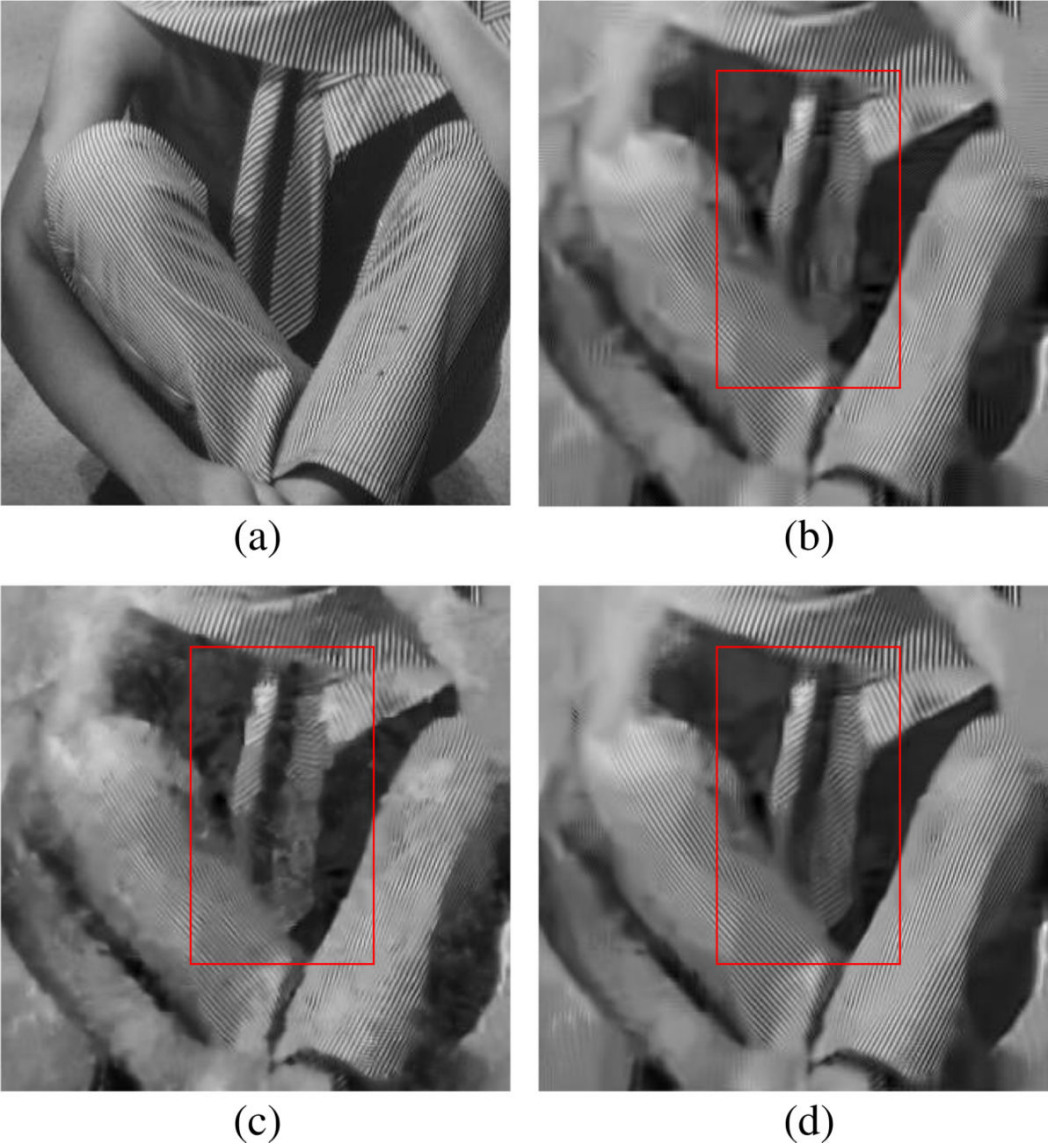
Comparison of denoised results by **b** BM3D, **c** BM3D-SAPCA, and **d** SA-BM1–3D, for the case of adding noise (σ = 100) on the original image shown in **a**

**Fig. 7:**
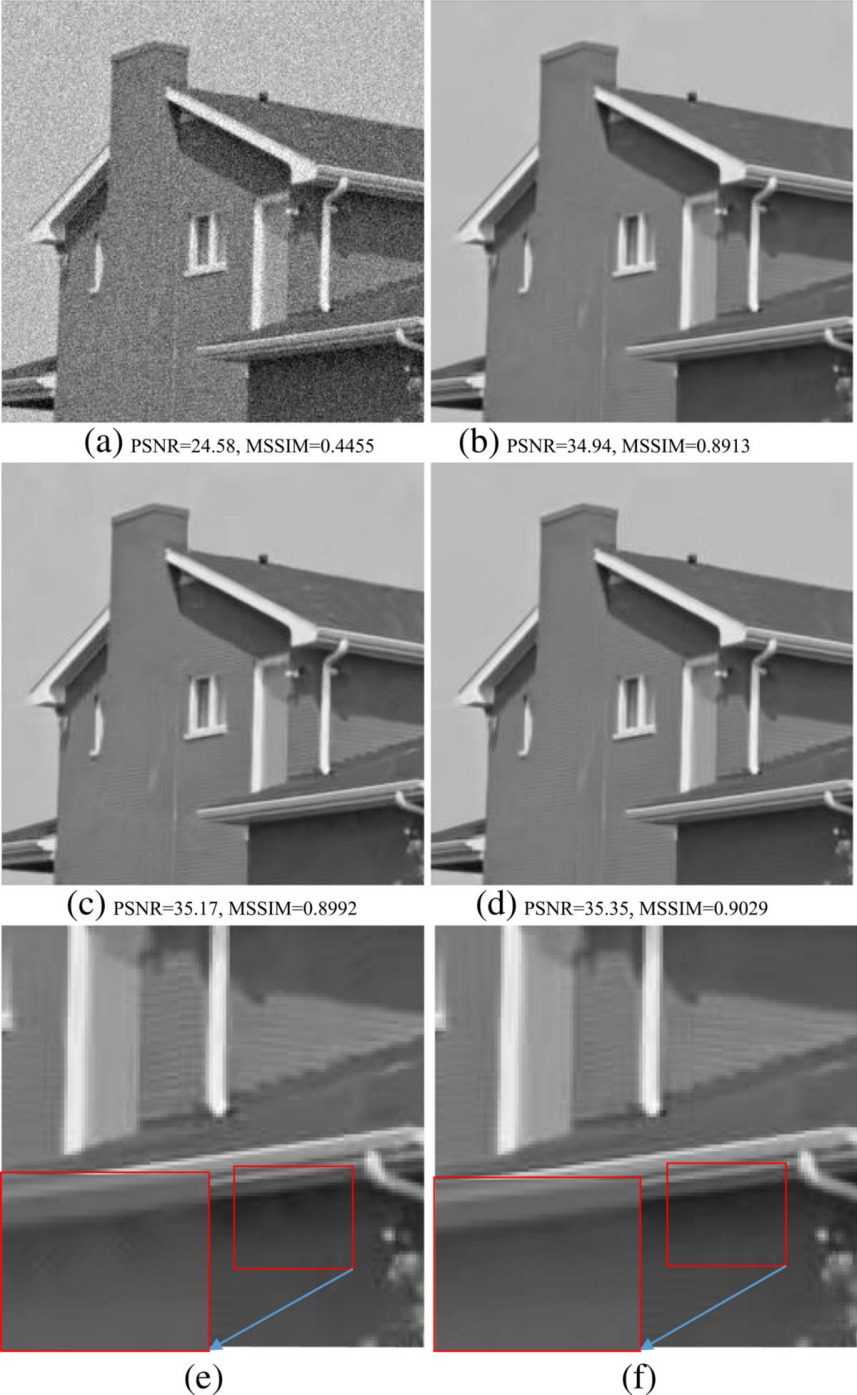
Comparison of denoised results by BM3D, BM3D-SAPCA, and SA-BM1–3D. **a** Noisy image (σ = 15), **b** BM3D, **c** BM3D-SAPCA, **d** SA-BM1–3D, **e** zoomed patch of (**c**), and **f** zoomed patch of (**d**)

**Fig. 8:**
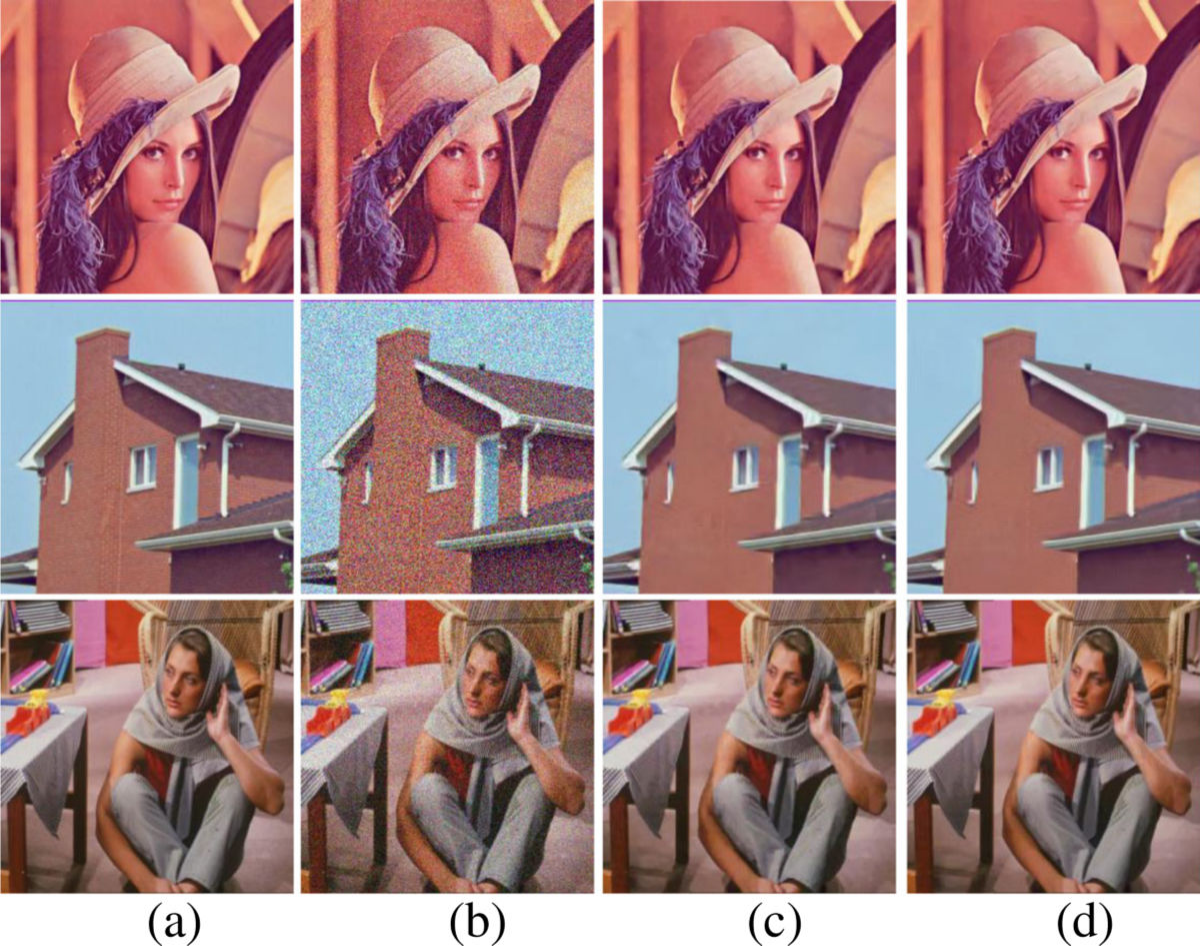
The denoised results comparison of color Lena and House images between BM3D and SA-BM1–3D (σ =35). **a** Original images, **b** noisy images, **c** denoised images by BM3D, and **d** denoised images by SA-BM1–3D

**Fig. 9:**
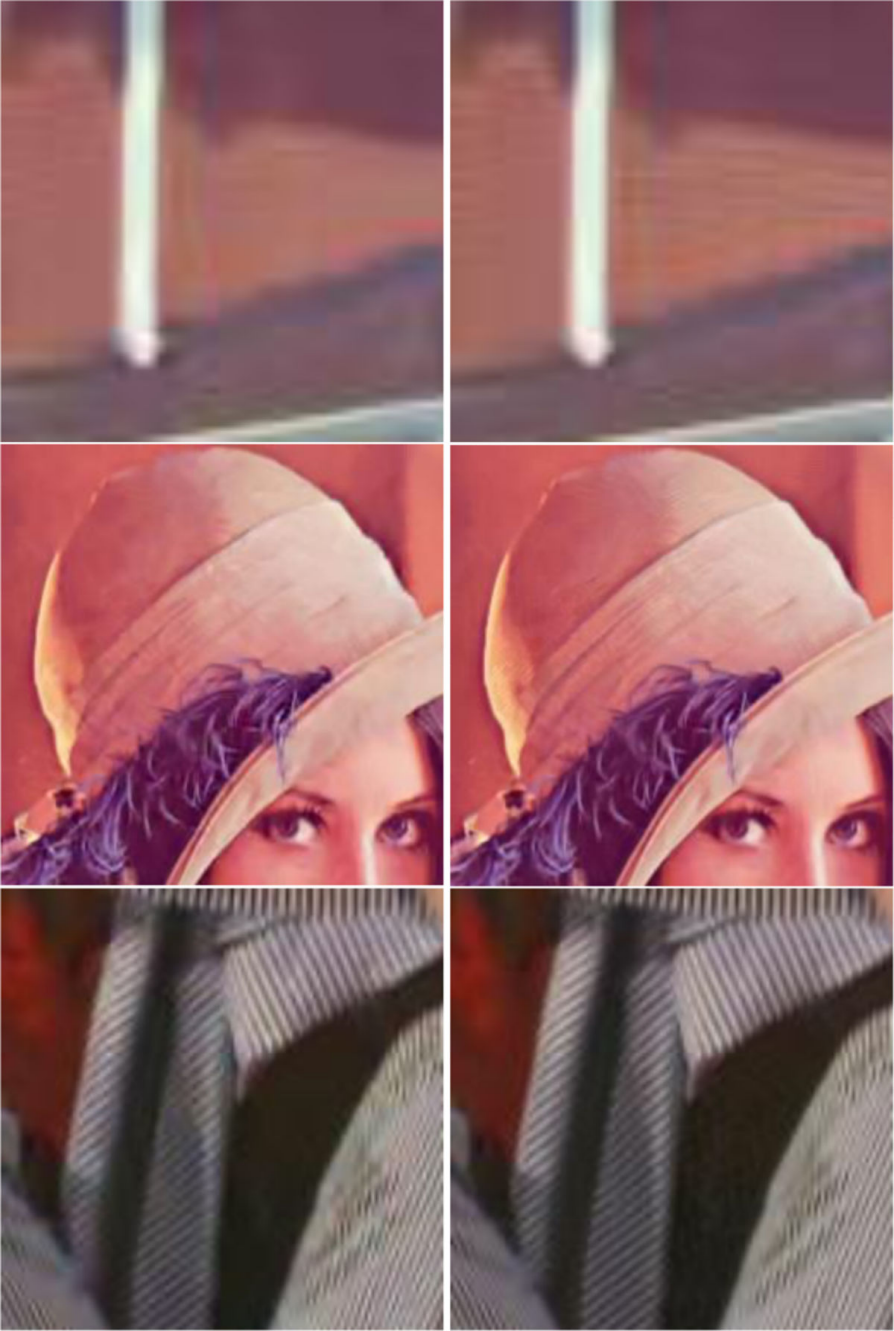
The denoised results comparison of color image fragments between BM3D and SA-BM1–3D (σ =35). Left: BM3D, right: SA-BM1–3D

We use the standard images provided in the BM3D website to conduct the denoising experiments. Table 2 shows the comparisons of PSNR values for the denoised results by proposed SA-BM1–3D, BM3D, EPLL in [8], SAIST in [29], LSSC in [34], and WNNM in [35]. On the other hand, Table 3 shows the comparisons of MSSIM values [44] for the denoised results by the BM3D, BM3D-SAPCA algorithm, and SA-BM1–3D. From these two tables, we can see that both PSNR and MSSIM values of SA-BM1–3D are consistently higher than those existing state-of-the-art algorithms, and are mostly higher than those of the BM3D-SAPCA and WNNM algorithms. Table 4 shows the comparison of PSNR for the denoised results on color images by the BM3D algorithm and SA-BM1–3D. From Table 4, we can see that the PSNR values of SA-BM1–3D are almost consistently higher than those of the BM3D algorithm. When the noise standard deviation is higher than 40, all the PSNR values of BM3D on both gray and color images are given by our previous improvement on BM3D [45]. Figure 6 shows the visual comparison of results by the BM3D algorithm, the BM3D-SAPCA algorithm, and SA-BM1–3D on a grayscale image. From this figure, we can see that when the noise level is relatively high, SA-BM1–3D still hardly introduces any artifacts, whereas the BM3D algorithm introduces a lot of periodic artifacts. The result by the BM3D-SAPCA algorithm is even worse than that obtained by the BM3D algorithm, since the step of determining adaptive shape in the BM3D-SAPCA algorithm fails for the case of strong *noise.* Figure 7 shows the comparison of three algorithms in denoising the House image. It can be seen that SA-BM1–3D can better preserve image details, but the BM3D-SAPCA algorithm introduces artifacts even with lower noise level. Figures 8 and 9 show the denoising results comparison between BM3D and SA-BM1–3D for color House, Lena, and Barbara images, as well as some zoomed in fragments, verifying the performance of SA-BM1–3D in the case of denoising color images. We can see from these two figures that SA-BM1–3D can better preserve image edge information than original BM3D method.

**Fig. 10:**
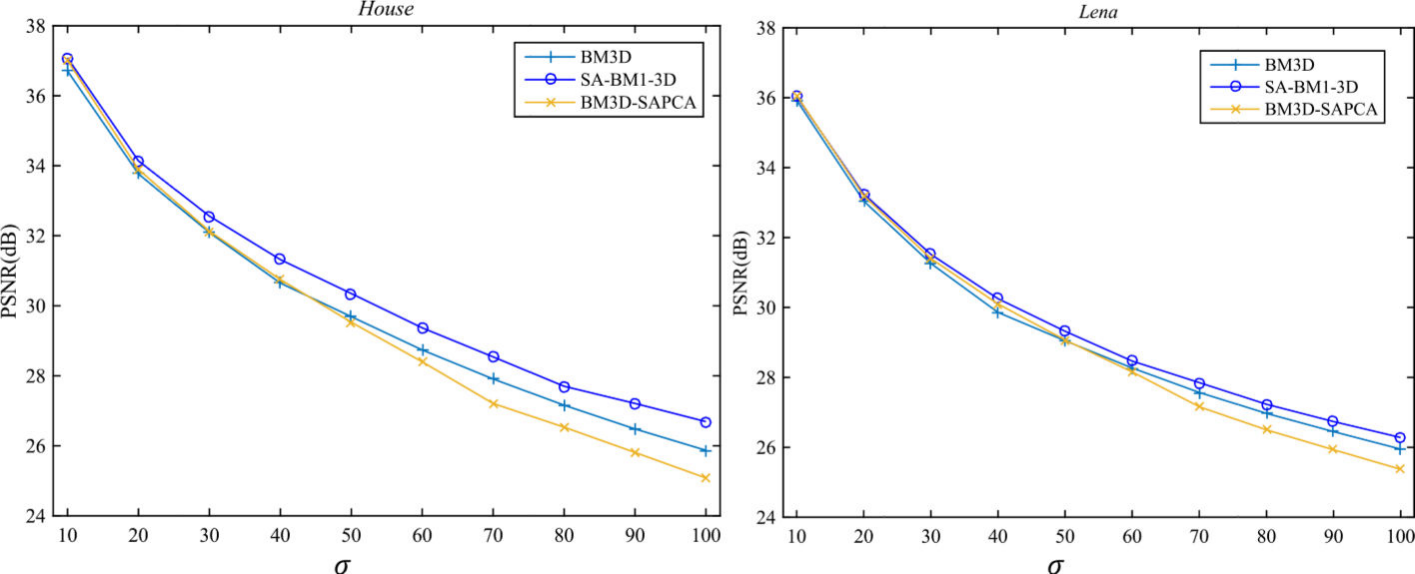
The denoised PSNR values comparison among BM3D, BM3D-SAPCA, and SA-BM1–3D for gray Lena and House images

Because SA-BM1–3D can better simulate human visual perception, it can achieve better image denoising results than the BM3D algorithm. In comparison to current state-of-the-art image denoising methods, such as BM3D-SAPCA, SA-BM1–3D is also generally competitive. Especially, by evaluating the denoising results with MSSIM, SA-BM1–3D can obtain better values than those by BM3D-SAPCA in most cases. We use Fig. 10 to intuitively show the denoised PSNRs comparison among BM3D, BM3D-SAPCA, and SA-BM1–3D for gray Lena and House images. We can see from Fig. 10 that SA-BM1–3D can achieve higher PSNR values than other two methods, BM3D-SAPCA can obtain higher PSNR values than BM3D in the low level noise case; however, it will be lower than BM3D when the noise level is relatively higher.

### 5.3 Computational complexity

In the total four stages of the proposed method, the number of operations per pixel is approximately

$$\frac{\left(S_{1}^{2}+N_{1}\right)N_{S}^{2}}{N_{\text{step}}^{2}}+\frac{S_{1}^{2}C_{T_{1D}}}{N_{\text{step}}^{2}}+\frac{\left(S_{2}^{2}+N_{2}\right)N_{S}^{2}}{N_{\text{step}}^{2}}\;\;+\frac{2S_{2}^{2}C_{T_{1D}}+2N_{2}C_{T_{2D}}}{N_{\text{step}}^{2}}+\frac{\left(S_{3}^{2}+N_{3}\right)N_{S}^{2}}{N_{\text{step}}^{2}}\;\;+\frac{S_{3}^{2}C_{T_{1D}}}{N_{\text{step}}^{2}}+\frac{\left(S_{4}^{2}+N_{4}\right)N_{S}^{2}}{N_{\text{step}}^{2}}\;\;+\frac{2S_{4}^{2}C_{T_{1D}}+2N_{4}C_{T_{2D}}}{N_{\text{step}}^{2}}+\frac{2C_{T_{2D}}}{N_{\text{step}}^{2}},$$

Where *C*_*Tl*D_ and *C*_*T*sD_ denote the number of arithmetic operations required for a ID and 2D transforms respectively.

The $\frac{\left(S_{i}^{2}+N_{i}\right)N_{S}^{2}}{N_{\text{step}}^{2}}$, *i* = 1, 2, 3, 4 denotes the number of block-matching operations, the $\frac{S_{i}^{2}C_{T_{1D}}}{N_{\text{step}}^{2}}$, *i* = 1, 3 denotes the number of 1D transform in stages 1 and 3, $\frac{2S_{i}^{2}C_{T_{1D}}+2N_{i}C_{T_{2D}}}{N_{\text{step}}^{2}}$, *i* = 2, 4 denotes the number of separable 3D transform in stages 2 and 4 for Wiener filtering. The last addend $\frac{2C_{T_{2D}}}{N_{\text{step}}^{2}}$ denotes the 2D-DCT transform number in stages 3 and 4 for morphological component analysis.

**Fig. 11:**
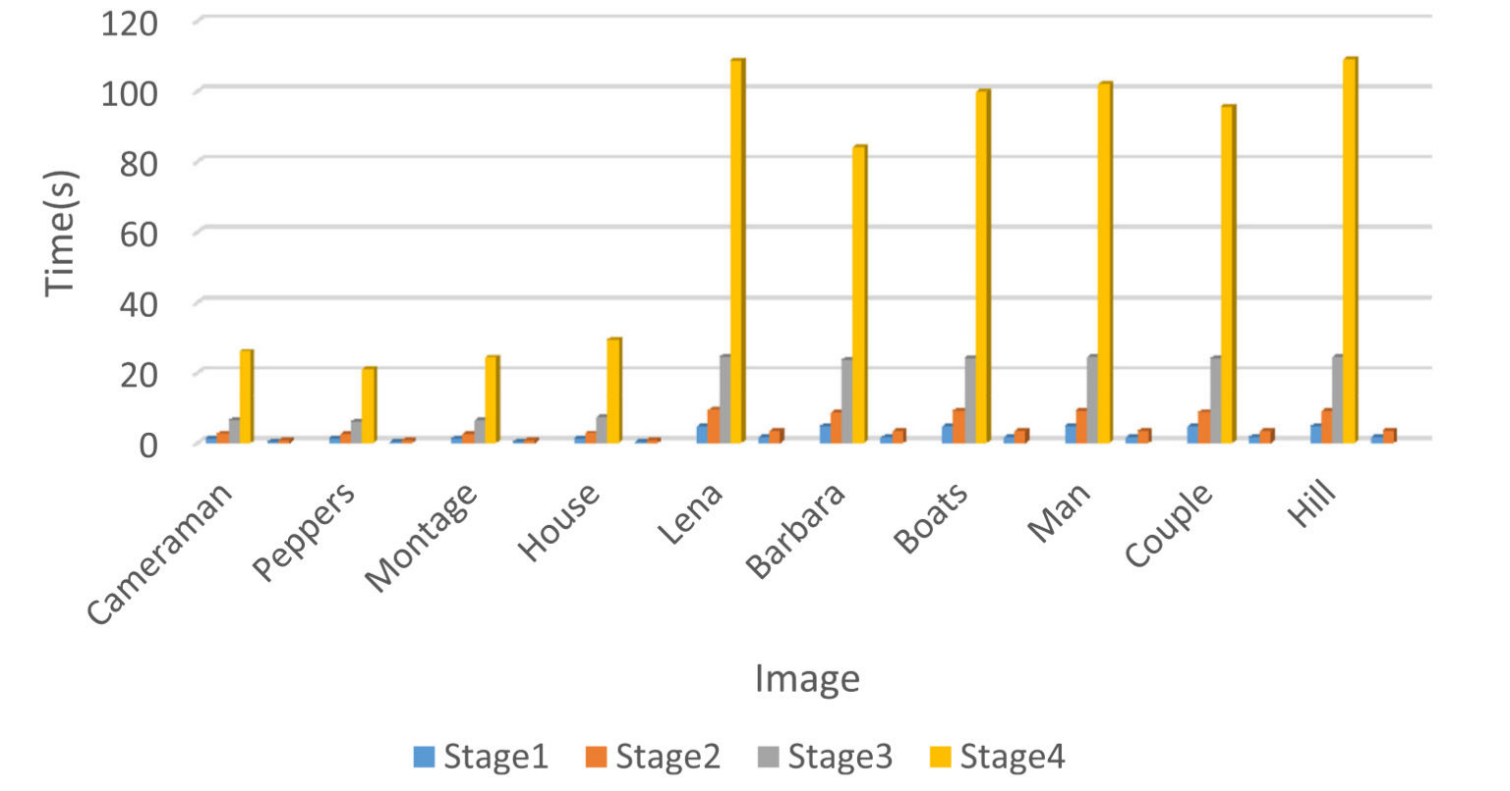
The time complexity comparison between BM3D and SA-BM1–3D for gray images

**Fig. 12:**
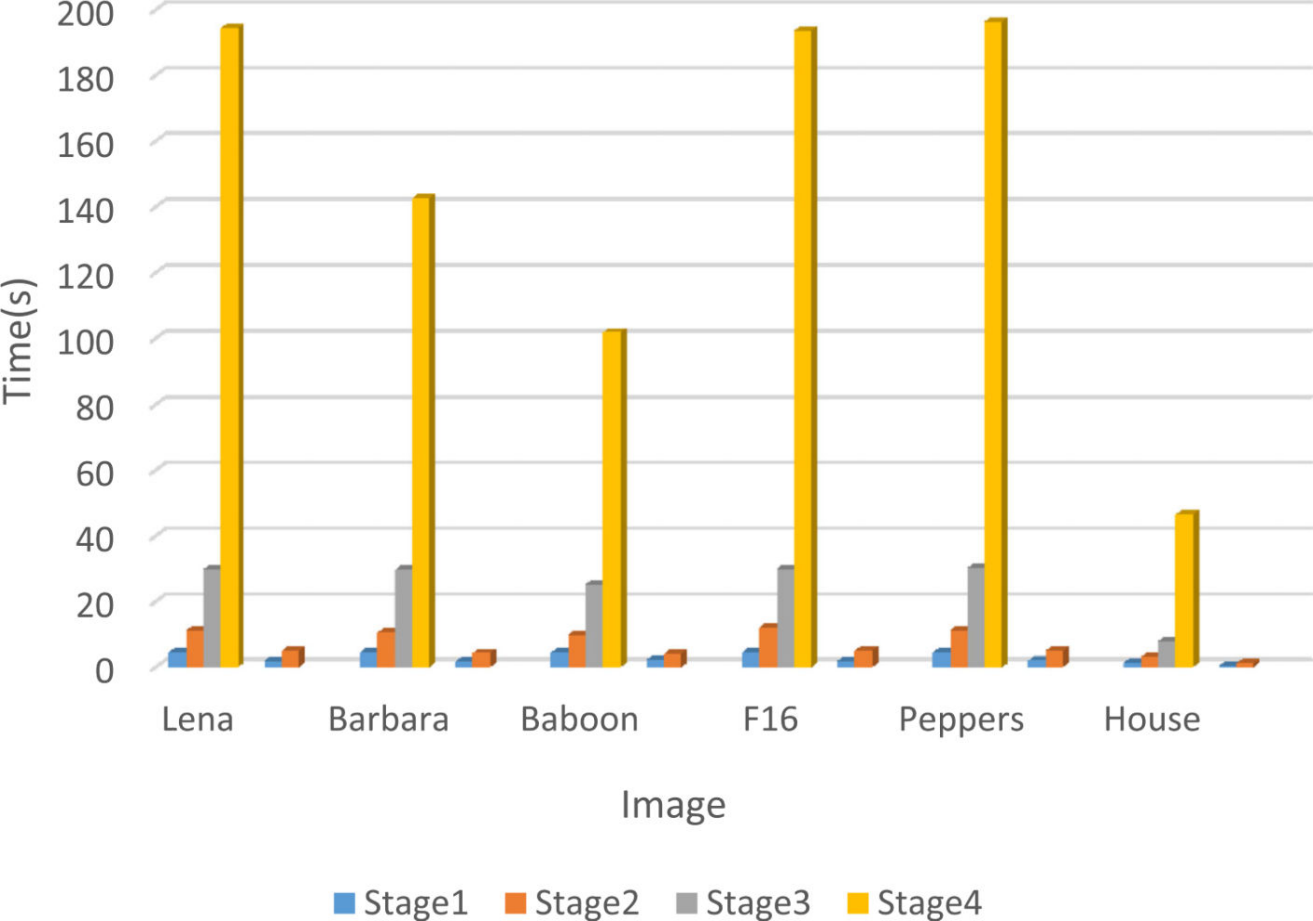
The time complexity comparison between BM3D and SA-BM1–3D for color images

Comparing the proposed method with BM3D, the computational complexity of the proposed method is higher than BM3D method indeed, the running time comparison between BM3D on a same computer and using the same MATLAB software is shown in Figs. 11 and 12; Fig. 11 shows the running time of SA-BM1–3D and BM3D on gray scale images with size 256 × 256 and 512 × 512 and Fig. 12 shows the running time for SA-BM1–3D and BM3D on color images of the size 256 × 256 and 512 × 512. We can see that the time complexity of the proposed method is much higher than BM3D, the reasons are mainly from the following two aspects: firstly, our programs did not optimized better than BM3D ones, for example, the computational complexity of the stage 1 in the proposed method is actually lower than the first stage in BM3D; however, the time complexity of the proposed method in this stage is much higher than BM3D. Secondly, the proposed method includes four stages but BM3D includes only two stages, especially, the size adaptive stages in the proposed method usually use bigger size image blocks than BM3D method, so it always increases the computational complexity. We will try to use the parallel computing or other strategies to lower the time complexity of the proposed method in the future work.

## 6 Conclusions

Based on human visual perception of noise in images, the image blocks in natural images, under denoising, can be divided into three morphological components, i.e., smooth, contour, and texture. Since the impact of noise is different in different regions, i.e., strongest in the smooth region followed by the texture and the contour regions, we propose using different parameter values (such as different thresholds and block sizes) on regions with different morphological components during image denoising. For example, we use a relatively large block size for the smooth regions since noise effect in the smooth regions seems strongest for human visual perception. On the other hand, we use the smallest block size for the contour regions, which are affected less by the noise.

Since DCT can depict the periodic signals very well, we use the AC energy of DCT coefficients to classify image blocks into three morphological components, i.e., smooth, texture, and contour. For the same block size, the image blocks with smooth component have the minimum AC energy followed by the image blocks with texture component and the image blocks with contour component. Experimental results have shown the robustness of our proposed algorithm to noise. Also, our proposed algorithm can achieve better denoising results than both BM3D and BM3D-SAPCA, in terms of PSNR and MSSIM values as well as visual inspection.

## Figures and Tables

**Table 1:** Parameter values used in SA-BM1–3D

σ	S1	S2	S3, S4	N1, N3	N2, N4	T1	T2	γ
0 ~ 2	4	8	8	8	32	3.0	1.0	1.1
3 ~ 9	5	8	8	8	32	3.0	1.0	1.1
10 ~ 44	8	8	8	8	32	3.0	0.8	1.5
45 ~ 64	9	9	8	16	32	3.0	0.6	2.0
65 ~ 84	10	10	8	16	32	3.0	0.6	2.0
≥ 85	15	11	8	16	32	3.0	0.4	2.5

**Table 2:** Comparison of PSNR values obtained by BM3D, BM3D-SAPCA, EPLL in [8], SAIST in [29], LSSC in [34], WNNM in [35], and proposed SA-BM1–3D

	BM3D	EPLL	SAIST	BM3D-SAPCA	LSSC	WNNM	SA-BM1–3D	BM3D	EPLL	SAIST	BM3D-SAPCA	LSSC	WNNM	SA-BM1–3D
	σ=10							Σ = 30						
C. man	34.18	34.02	34.30	**34.59**	34.24	34.44	34.40	28.64	28.36	28.36	28.90	28.63	28.80	**28.92**
House	36.71	35.75	36.66	37.01	36.95	36.95	**37.08**	32.09	30.99	32.30	32.13	32.41	32.52	**32.56**
Peppers	34.68	34.54	34.82	34.94	34.80	**34.95**	34.84	29.28	29.16	29.24	29.51	29.25	29.49	**29.53**
Montage	37.35	36.49	37.46	**37.85**	37.26	37.84	37.71	31.37	30.17	31.06	31.92	31.10	31.65	**31.98**
Lena	35.93	35.58	35.90	**36.07**	35.83	36.03	36.04	31.26	30.79	31.27	31.40	31.18	31.43	**31.52**
Barbara	34.98	33.61	35.24	35.10	34.98	**35.51**	34.91	29.81	27.57	30.14	30.12	29.60	**30.31**	30.10
Boats	33.92	33.66	33.91	**34.10**	34.01	34.09	34.06	29.12	28.89	28.98	29.22	29.06	29.24	**29.29**
Man	33.98	33.97	34.12	**34.25**	34.10	34.23	34.16	28.86	28.83	28.81	29.04	28.87	29.00	29.01
Couple	34.04	33.85	33.96	**34.17**	34.01	34.14	34.16	28.87	28.62	28.72	28.95	28.77	28.98	**29.07**
Hill	33.62	33.48	33.65	**33.83**	33.66	33.79	33.80	29.16	28.90	29.06	29.23	29.09	29.25	**29.31**
	σ=50							σ = 100						
C.man	26.12	26.02	26.15	26.58	26.35	26.42	**26.67**	23.07	22.86	23.09	22.88	23.15	23.36	**23.56**
House	29.69	28.76	30.17	29.53	29.99	30.32	**30.35**	25.87	25.19	26.53	25.08	25.71	26.68	**26.69**
Peppers	26.68	26.63	26.73	27.00	26.79	26.91	**27.06**	23.39	23.08	23.32	23.24	23.20	23.46	**23.63**
Montage	27.9	27.17	28.00	28.59	28.10	28.27	**28.88**	23.89	23.42	23.98	23.96	23.77	24.16	**24.65**
Lena	29.05	28.42	29.01	29.07	28.95	29.24	**29.32**	25.95	25.30	25.93	25.37	25.96	26.20	**26.28**
Barbara	27.23	24.82	27.51	27.49	27.03	**27.79**	27.64	23.62	22.14	24.07	23.09	23.54	**24.37**	24.36
Boats	26.78	26.65	26.63	26.89	26.77	26.97	**27.05**	23.97	23.71	23.80	23.69	23.87	24.10	**24.13**
Man	26.81	26.72	26.68	**26.94**	26.72	**26.94**	**26.94**	24.22	24.07	24.01	23.96	23.98	24.36	**24.38**
Couple	26.46	26.24	26.30	26.49	26.35	26.65	**26.70**	23.51	23.32	23.21	23.21	23.27	23.55	**23.69**
Hill	27.19	26.96	27.04	27.20	27.14	27.34	**27.37**	24.58	24.43	24.29	24.27	24.47	24.75	**24.78**

Data in bold are the best results

**Table 3:** Comparison of MSSIM values obtained by BM3D, BM3D-SAPCA, and SA-BM1–3D. In each cell, the upper number is for BM3D, the middle number is for BM3D-SAPCA, while the lower number is for SA-BM1–3D

σ/MSSIM	C. man	House	Peppers	Montage	Lena	Barbara	Boats	Man	Couple	Hill
5	0.9620	0.9571	0.9558	0.9823	0.9444	0.9647	0.9389	0.9543	0.9512	0.9427
	0.9629	0.9595	0.9568	0.9824	0.9461	0.9659	0.9438	**0.9565**	0.9529	**0.9455**
	**0.9638**	**0.9604**	**0.9573**	**0.9826**	**0.9478**	**0.9664**	**0.9443**	0.9559	**0.9535**	**0.9455**
10	0.9319	0.9218	0.9282	0.9679	0.9166	0.9421	0.8878	0.9076	0.9094	0.8834
	**0.9352**	0.9290	0.9287	0.9690	0.9183	**0.9433**	0.8924	**0.9125**	0.9117	0.8896
	0.9345	**0.9313**	**0.9296**	**0.9694**	**0.9197**	0.9428	**0.8928**	0.9121	**0.9135**	**0.8906**
15	0.9014	0.8913	0.9068	0.9539	0.8956	0.9233	0.8539	0.8672	0.8765	0.8387
	**0.9096**	0.8992	0.9068	0.9563	0.8982	**0.9258**	0.8574	**0.8740**	0.8784	**0.8455**
	0.9070	**0.9029**	**0.9093**	**0.9578**	**0.8999**	**0.9258**	**0.8589**	0.8728	**0.8802**	0.8452
20	0.8755	0.8726	0.8868	0.9404	0.8772	0.9054	0.8259	0.8333	0.8476	0.8040
	**0.8857**	0.8763	0.8874	0.9430	0.8809	0.9094	0.8290	**0.8407**	0.8491	0.8094
	0.8831	**0.8799**	**0.8909**	**0.9451**	**0.8830**	**0.9103**	**0.8311**	0.8388	**0.8520**	**0.8102**
25	0.8544	0.8589	0.8676	0.9262	0.8607	0.8874	0.8014	0.8047	0.8204	0.7748
	**0.8642**	0.8607	0.8690	0.9297	0.8650	0.8942	0.8039	**0.8111**	0.8215	0.7788
	0.8618	**0.8648**	**0.8732**	**0.9335**	**0.8677**	**0.8951**	**0.8067**	0.8094	**0.8266**	**0.7808**
30	0.8375	0.8480	0.8505	0.9114	0.8449	0.8687	0.7795	0.7802	0.7947	0.7504
	**0.8442**	0.8495	0.8520	0.9175	0.8505	0.8780	0.7824	**0.7860**	0.7962	0.7529
	0.8437	**0.8548**	**0.8564**	**0.9222**	**0.8540**	**0.8798**	**0.7852**	0.7833	**0.8030**	**0.7549**
35	0.8218	0.8372	0.8340	0.8962	0.8305	0.8482	0.7593	0.7579	0.7708	0.7283
	0.8276	0.8381	0.8360	0.9037	0.8373	0.8610	0.7617	**0.7633**	0.7730	0.7299
	**0.8289**	**0.8466**	**0.8406**	**0.9108**	**0.8412**	**0.8637**	**0.7655**	0.7606	**0.7801**	**0.7328**
40	0.8057	0.8256	0.8158	0.8816	0.8152	0.8225	0.7387	0.7374	0.7469	0.7069
	0.8126	0.8301	0.8222	0.8900	0.8247	0.8448	0.7433	**0.7434**	0.7492	0.7096
	**0.8159**	**0.8385**	**0.8267**	**0.8995**	**0.8291**	**0.8466**	**0.7466**	0.7405	**0.7586**	**0.7126**
50	0.7824	0.8122	0.7936	0.8614	0.7994	0.7946	0.7053	0.7056	0.7068	0.6747
	0.7868	0.8078	0.7942	0.8643	0.8014	0.8088	0.7081	**0.7107**	0.7079	0.6756
	**0.7939**	**0.8274**	**0.8016**	**0.8770**	**0.8107**	**0.8171**	**0.7143**	0.7093	**0.7207**	**0.6803**
60	0.7626	0.7941	0.7698	0.8365	0.7795	0.7589	0.6767	0.6786	0.6715	0.6470
	0.7613	0.7855	0.7688	0.8361	0.7788	0.7691	0.6773	0.6812	0.6698	0.6456
	**0.7761**	**0.8116**	**0.7758**	**0.8540**	**0.7911**	**0.7797**	**0.6846**	**0.6814**	**0.6843**	**0.6514**
70	0.7424	0.7747	0.7477	0.8116	0.7603	0.7261	0.6527	0.6548	0.6406	0.6226
	0.7226	0.7448	0.7339	0.7926	0.7401	0.7161	0.6409	0.6460	0.6237	0.6131
	**0.7612**	**0.7999**	**0.7629**	**0.8414**	**0.7768**	**0.7508**	**0.6602**	**0.6604**	**0.6517**	**0.6265**
80	0.7246	0.7557	0.7276	0.7886	0.7426	0.6966	0.6305	0.6338	0.6124	0.6009
	0.6933	0.7228	0.7079	0.7574	0.7142	0.6711	0.6180	0.6206	0.5944	0.5873
	**0.7438**	**0.7836**	**0.7467**	**0.8232**	**0.7613**	**0.7229**	**0.6375**	**0.6403**	**0.6196**	**0.6041**
90	0.7079	0.7380	0.7068	0.7667	0.7261	0.6687	0.6109	0.6154	0.5882	0.5824
	0.6696	0.7021	0.6933	0.7377	0.6949	0.6390	0.5965	0.5986	0.5701	0.5657
	**0.7343**	**0.7730**	**0.7173**	**0.7953**	**0.7476**	**0.7076**	**0.6233**	**0.6243**	**0.6069**	**0.5905**
100	0.6924	0.7203	0.6881	0.7474	0.7090	0.6430	0.5936	0.5978	0.5665	0.5650
	0.6445	0.6760	0.6726	0.7131	0.6747	0.6088	0.5789	0.5793	0.5472	0.5492
	**0.7207**	**0.7641**	**0.7063**	**0.7865**	**0.7367**	**0.6890**	**0.6072**	**0.6112**	**0.5828**	**0.5740**

Data in bold are the best results

**Table 4:** Comparison of PSNR values obtained by BM3D and SA- BM1–3D on color images. In each cell, the upper number is for BM3D while the lower number is for SA-BM1–3D

σ/PSNR	Lena	Peppers	Baboon	F16	House	Barbara
5/34.16	37.82	36.82	35.25	39.68	38.97	41.42
	**37.97**	**37.04**	**35.34**	**39.80**	**39.12**	**41.45**
10/28.14	35.22	33.78	30.64	36.68	36.23	37.84
	**35.31**	**33.94**	**30.81**	**36.80**	**36.40**	**37.88**
15/24.61	33.94	32.60	28.39	34.99	34.85	35.86
	**34.02**	**32.67**	**28.58**	**35.15**	**35.07**	**35.89**
20/22.11	33.02	31.83	26.97	33.77	33.84	34.45
	**33.10**	**31.86**	**27.17**	**33.94**	**34.09**	**34.48**
25/20.18	32.27	31.20	25.95	32.78	33.03	33.31
	**32.35**	**31.22**	**26.14**	**32.99**	**33.29**	**33.38**
30/18.59	31.59	30.61	25.14	31.93	32.34	32.26
	**31.79**	**30.71**	**25.31**	**32.28**	**32.66**	**32.58**
35/17.25	30.91	30.00	24.46	31.13	31.58	31.10
	**31.26**	**30.23**	**24.68**	**31.63**	**32.12**	**31.78**
50/14.16	29.88	28.93	23.15	29.79	30.47	29.91
	**30.02**	**29.05**	**23.28**	**30.05**	**30.82**	**30.14**
75/10.64	28.36	27.38	21.76	27.97	28.69	27.80
	**28.51**	**27.47**	**21.84**	**28.07**	**29.14**	**28.06**
100/8.14	27.11	26.06	20.72	26.42	26.70	26.10
	**27.34**	**26.30**	**20.88**	**26.67**	**27.18**	**26.61**

Data in bold are the best results

## Abbreviations

AC: Alternating current
AWGN: Additive white Gaussian noise
BM1D: Block matching and 1D filtering
BM3D: Block matching and 3D filtering
BM3D- SAPCA: Shape adaptive principal component analysis block matching and 3D filtering
DCT: Discrete cosine transform
EPLL: Expected patch log likelihood
HOSVD: Higher order singular value decomposition
LSSC: Learned simultaneous sparse coding
MSSIM: Mean structural similarity
NLM: Non-local means
PCA: Principal component analysis
PSNR: Peak signal to noise ratio
SA-BM1–3D: Size-adaptive block matching 1D and 3D filtering
SA-BM3D: Shape-adaptive block matching and 3D filtering
SA-DCT: Shape-adaptive discrete cosine transform
SAIST: Spatially adaptive iterative singular-value thresholding
WNNM: Weighted nuclear norm minimization
